# Potential Inhibitors for Isocitrate Lyase of *Mycobacterium tuberculosis* and Non-*M. tuberculosis*: A Summary

**DOI:** 10.1155/2015/895453

**Published:** 2015-01-08

**Authors:** Yie-Vern Lee, Habibah A. Wahab, Yee Siew Choong

**Affiliations:** ^1^Institute For Research in Molecular Medicine, Universiti Sains Malaysia, 11800 Minden, Penang, Malaysia; ^2^Pharmaceutical Design and Simulation Laboratory, School of Pharmaceutical Sciences, Universiti Sains Malaysia, 11800 Minden, Penang, Malaysia; ^3^Natural Product and Drug Discovery Centre, Malaysian Institutes of Pharmaceuticals and Nutraceuticals, National Institutes of Biotechnology Malaysia, Ministry of Science, Technology and Innovation, Block 5-A, Halaman Bukit Gambir, 11700 Penang, Malaysia; ^4^ADAPT Research Cluster, Centre for Research Initiatives, Clinical & Health Sciences, Universiti Sains Malaysia, 16150 Kubang Kerian, Kelantan, Malaysia

## Abstract

Isocitrate lyase (ICL) is the first enzyme involved in glyoxylate cycle. Many plants and microorganisms are relying on glyoxylate cycle enzymes to survive upon downregulation of tricarboxylic acid cycle (TCA cycle), especially *Mycobacterium tuberculosis* (MTB). In fact, ICL is a potential drug target for MTB in dormancy. With the urge for new antitubercular drug to overcome tuberculosis treat such as multidrug resistant strain and HIV-coinfection, the pace of drug discovery has to be increased. There are many approaches to discovering potential inhibitor for MTB ICL and we hereby review the updated list of them. The potential inhibitors can be either a natural compound or synthetic compound. Moreover, these compounds are not necessary to be discovered only from MTB ICL, as it can also be discovered by a non-MTB ICL. Our review is categorized into four sections, namely, (a) MTB ICL with natural compounds; (b) MTB ICL with synthetic compounds; (c) non-MTB ICL with natural compounds; and (d) non-MTB ICL with synthetic compounds. Each of the approaches is capable of overcoming different challenges of inhibitor discovery. We hope that this paper will benefit the discovery of better inhibitor for ICL.

## 1. Introduction 

### 1.1. Isocitrate Lyase

According to the ENZYME nomenclature database, isocitrate lyase (ICL; E.C. number 4.1.3.1) is also known as isocitrase, isocitritase, isocitratase, and isocitrate glyoxylate-lyase [1]. ICL can be found in Archaea, bacteria, fungi, nematodes, plants, and protists. In general, ICL plays an important role in seed germination in higher plants, microbial pathogenicity, and survival.

**Figure 1:**
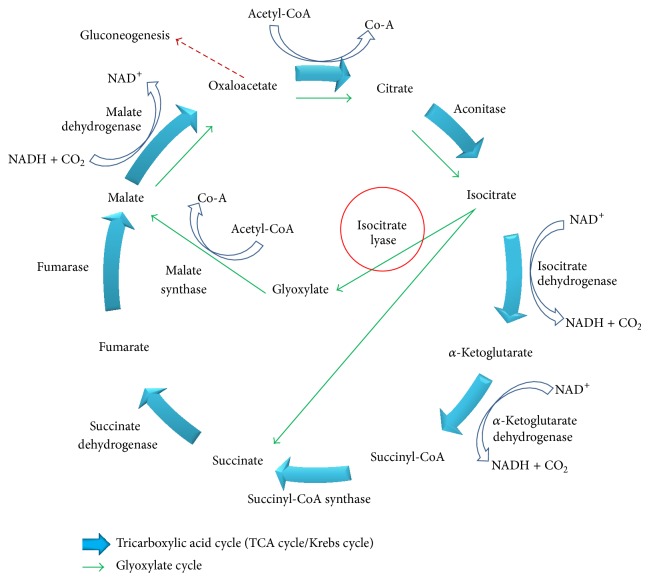
The general scheme for tricarboxylic acid (TCA) cycle (blue bold arrows) and glyoxylate cycle (green arrows). Isocitrate lyase (ICL, circled in red) is the first enzyme involved in the glyoxylate cycle. Oxaloacetate might leave the cycle as the substrate of gluconeogenesis (red dash arrow).

Glyoxylate cycle is an alternative pathway to generate energy when tricarboxylic acid cycle (TCA cycle or Krebs cycle) is downregulated upon oxygen and nutrient depletion [2]. When most of the TCA enzymes are suppressed, glyoxylate enzymes will be upregulated. By utilizing glyoxylate cycle, some beta oxidation steps in TCA cycle are bypassed. The early phase of glyoxylate cycle resembles the TCA cycle (Figure 1), and the acetyl-CoA is the only substrate for both TCA and glyoxylate cycle. However, the source of precursor, acetyl-CoA, is different for respective cycle. Carbohydrate undergoes glycolysis to generate the acetyl-CoA while lipid undergoes beta-oxidation to generate acetyl-CoA. The point of differentiation for these two cycles begins when acetyl-CoA is converted to isocitrate. In glyoxylate cycle, two important enzymes are required: ICL and malate synthase (MS). ICL carries the function to reversibly cleave the isocitrate to glyoxylate and succinate while MS will convert glyoxylate into malate by adding an acetyl group. Earlier study showed that during downregulation of TCA cycle, the inhibition of ICL is fatal for MTB [3].

To date, a total of seven ICL crystal structures were solved for five different microorganisms:* Aspergillus nidulans *[PDB id: 1DQU [4]],* Mycobacterium tuberculosis *[PDB id: 1F61 [5]; 1F8I [5]; 1F8M [5]],* Escherichia coli *[PDB id: 1IGW [6]],* Burkholderia pseudomallei* [PDB id: 3I4E (paper unpublished)],* Brucella melitensis* [PDB id: 3EOL, 3P0X, 3OQ8, and 3E5B (paper unpublished)], and* Yersinia pestis *[PDB id: 3LG3 (paper unpublished)]. However, no plant ICL structure has been solved. Only a handful of crystal structures were obtained for ICL as it is likely due to the difficulty in controlling the evaporation rate of crystallizing solution [7].

### 1.2. MTB ICL Related Studies

**Figure 2:**
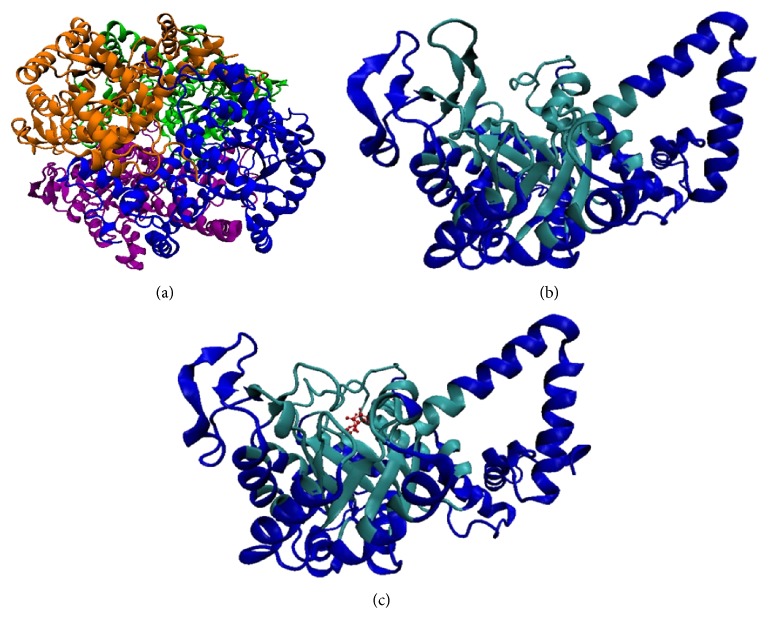
The structure of* Mycobacterium tuberculosis* isocitrate lyase in ribbon representation. (a) ICL tetramer with each subunit is represented by different colors [5]. (b) ICL monomer with active site (in cyan color) in “open conformation” and (c) ICL monomer with active site (in cyan color) in “close conformation” substrates (succinate and glyoxylate) are bound in the active site with red CPK representation.

The structure of MTB ICL (Figure 2) was solved by Sharma et al. [5] (PDB id: 1F61, 1F8I, 1F8M). Current available data showed that ICL is stable as a dimer but it will only be functional in a tetrameric form [5, 8]. Each subunit has an unusual *α*/*β* barrel as its largest core domain which consists of eight *α*-helixes and *β*-strands, respectively. An extra *α*-helix was projected out from the barrel of each subunit with another two ensuing *α*-helices which are involved in the interaction with neighboring subunit. On top of the barrel, there is an important small *β*-domain with several active side residues. 1F61 is a ligand-free ICL which has “open conformation” active site. 1F8I and 1F8M are ICL that bind succinate/glyoxylate and pyruvate, respectively. Ligand binding leads to conformational change triggering the ICL active site to shift into a “close conformation.” The catalytic mechanism of forming isocitrate from glyoxylate, succinate, and vice versa was mentioned. Glyoxylate was proposed to bind with ICL first before succinate as the former buried deeper than the latter. As per the cleavage mechanism of isocitrate to glyoxylate and succinate, the authors proposed that isocitrate C–C bond cleaved via Claisen condensation. However, the cleavage information from isocitrate-ICL complex structure is needed, which is unavailable at the moment to further confirm the abovementioned hypothesis.

The potential of ICL as a drug target has been proven by several studies. According to Dunn et al., [9] ICL gene is not found in mammals; therefore theoretically it is safe if a drug targeting at ICL is administrated to human. Muñoz-Elías and McKinney [3] showed that two types of MTB ICLs (ICL1: prokaryotic-like isoform and ICL2: eukaryotic-like isoform) are jointly required for MTB survival. They showed that absence of either one ICL isoform will not harm the survival of MTB but absence of both isoforms will cause MTB to be eliminated from the host lungs. These two ICL isoforms are coded by* icl *gene (ICL1) and* ace A* gene (ICL2), respectively [10]. Current research involving ICL is mainly focused on ICL1 (including the solved structure of ICL). Another isoforms, the* ace A* gene, is however less active compared to* icl* gene and is not expressed in all mycobacterium strain [11]. Furthermore, Gould et al. [12] reported that MTB ICL1 has dual roles, in both glyoxylate cycle and methylcitrate cycle. Methylcitrate cycle is a mechanism that removes propionyl-CoA, a toxic by-product of lipid beta-oxidation. Three enzymes involved in methylcitrate cycle are methylcitrate synthase, methylcitrate dehydrogenase, and 2-methylisocitrate lyase (MCL). It was found that MTB only produce methylcitrate synthase and methylcitrate dehydrogenase but not MCL. The function of MCL was carried out by ICL1, making it more important than expected [12].

Singh and Ghosh highlighted that both ICL and isocitrate dehydrogenase (IDH) compete for the same substrate, which is the isocitrate [13]. A new approach to inhibit ICL by increasing the IDH's concentration was demonstrated. This is also indicative that IDH has higher affinity towards isocitrate compared to ICL. However, IDH-kinase counter inhibits IDH action, thus allowing ICL to bind isocitrate and proceed through the glyoxylate pathway. IDH-kinase was also proposed as a potential drug target for IDH-kinase inhibition which could lead to ICL inhibition [13].

Till date, several ICL inhibitors like itaconate [14], 3-nitropropionate [15], and 3-bromopyruvate [16] have been identified. However, these inhibitors are not suitable as drug due to their toxicity and their ability to inhibit key metabolism enzymes* in vivo*. For example, itaconate was suspected to cause hypertonicity of blood pressure in cats [17] and affects the growth of rats [18]; 3-nitropropionate was found to cause neurotoxicity [19] whereas 3-bromopyruvate seems to be an energy blocker [20, 21]. To date, various research groups are seeking new potential inhibitor for ICL. The inhibitors screening approaches are similar but targeted the ICL from different species and are summarized in next sections.

## 2. Discovery of Isocitrate Lyase Potential Inhibitors from Different Approaches

Among all species, ICL of* Mycobacterium tuberculosis* (MTB) gains the most attention as it is related to tuberculosis, which had infected one-third of the world population [22]. As MTB survives in both active and inactive (dormant) phase with different metabolic pathway, identification of common drug target for these two phases that utilize different metabolic pathways is rather difficult. Therefore, MTB drug target study for each respective phase is important [23]. Active MTB operates TCA cycle, using sugar as main carbon source to generate energy. However, phagocytosis of MTB by macrophage causes oxygen and nutrient depletion, causing MTB to enter its dormant phase. This causes a massive metabolic shunt and downregulates TCA enzymes [24]. In order to maintain MTB viability, glyoxylate enzymes will be upregulated in order to continue generating energy from an alternative carbon source, namely, lipids. Other than MTB, similar enzyme regulation in TCA and glyoxylate cycle has been observed in other opportunistic pathogens such as bacterium (*Pseudomonas aeruginosa* [25]), fungus (*Candida albicans* [26],* Magnaporthe grisea* [27], and* Leptosphaeria maculans* [28]), and nematode (*Caenorhabditis elegans *and* Ascaris suum *[29]). Due to the importance of ICL during glyoxylate cycle towards various microorganisms, ICL has been studied intensively.

MTB ICL is one of the most difficult organism/enzyme to study as it grows slow and has a higher risk of infection. Biosafety level three training and facility are necessary to study live MTB. Therefore, several strategies to screen ICL inhibitor were derived. The source of ICL used for ICL inhibitor studies is categorized into MTB and non-MTB ICL, respectively. The source of inhibitor however could either be natural or synthetic compounds (Table 1).

### 2.1. MTB ICL with Natural Compound

First high-throughput screening (HTS) report on natural compound using MTB ICL was released on 2006 by Bai and coworkers [30]. A total of 465 traditional Chinese medicines were screened against MTB ICL. Two extracts,* Zingiber officinale* (IC_50_ of 47.7 *μ*g/mL) and* Illicium verum *(IC_50_ of 18.2 *μ*g/mL), were reported to have inhibitory effect. In 2010, a subsequent article was released, reporting a novel lead compound I2906 (1-ethyl-4-hydroxy-2-oxo-N′-tridecanoyl-1,2-dihydroquinoline-3-carbohydrazide) with an IC_50_ of 134.4 *μ*g/mL [31]. Chelerythrine extract from the plant* Chelidonium majus* was also reported as a potential drug which causes fivefold decrease in ICL gene expression [32].

### 2.2. MTB ICL with Synthetic Compound

The common strategy to obtain synthetic compounds is to obtain analog or derivatives of existing potential inhibitor, regardless of MTB or non-MTB ICL. The ultimate goal of this strategy is not only to look for new inhibitors, but also to improve the inhibitory potential of existing ones. A thorough review for synthetic compounds targeting MTB ICL is available [33, 34]. The review articles have reviewed the synthetic compounds such as pthalazinyl derivatives [35], phthalazin-4-ylacetamides [36], 5-nitro-2-furoic acid hydrazones with furan-2-carbaldehyde [37], 5-Nitro-2,6-dioxohexahydro-4-pyrimidinecarboxamides [38], isatinyl thiosemicarbazones derivatives [39], and 3-nitropropionamide derivatives [40] with 45–61%, 40.62–66%, 86.8%, 45.7%, and 63.44% inhibition at 10 *μ*M and IC_50_ of 0.1 *μ*M, respectively.

Other than derivative synthesis, other categories of synthetic compound such as DNAzymes [41], Mannich bases [42], peptide inhibitors [43, 44], and pyruvate-isoniazid analog with their copper complex [45] also gained some attention in the crowd. In 2005, the concept of silencing the* icl* gene by DNAzymes was introduced. The study showed that several designed DNAzymes (DZ1, DZ3, DZ4, and DZ5) were capable of specifically cleave ICL mRNA, which leads to interruption of ICL expression in macrophage. However, DNAzyme did not show any effect toward* in vitro* MTB growth when combined with another inhibitory drug such as isoniazid [41]. Later Mannich base, Ydcm67, was reported as one of the best inhibitors (57.4% inhibition at 0.05 mg/mL) out of 124 Mannich bases screened, but no* in vivo* data is shown [42]. For peptide inhibitors, 38.82–47.92% inhibition rate was obtained but the authors concluded that these peptide inhibitors might be too small in size and might face some drug delivery issue [43]. However, in 2013, the subsequent article has made some optimization for the peptide inhibitors. Liu et al. first screen a phage peptide library against MTB ICL to obtain 29 potential inhibitors and perform molecular docking simulation to confirm the hit. Liu and coworker managed to synthesize the 12 peptides out of 29 that were shown to have successfully docked into ICL crystal structure using Ligand Fit module of Discovery Studio 2.1 software (PDB id 1F8M) and one of the peptides has obtained as high as IC_50_ of 126 *μ*M in bioassay [44]. Regarding pyruvate-isoniazid analog with its copper complex, preliminary result of 6–92% ICL inhibition still requires further investigation to its inhibitory mechanism [45].

### 2.3. Non-MTB ICL with Natural Compound

Natural compounds are well known in certain parts of the world with remedial potential. The search for ICL inhibitors has also expanded to screen natural products and to date, marine sponges and algae are the most common species explored. Several ICL inhibitors from these sources were discovered using ICL of* Magnaporthe grisea* and* Candida albicans*. Compounds discovered through* M. grisea* are halisulfate 1 (*Hippospongia *spp.) [46, 47] and bromophenol (*Odonthalia corymbifera*) [27] with IC_50_ of 12.6 *μ*M and 2.0–2.8 *μ*M, respectively, whereas, compounds discovered through* C. albicans* ICL are meroditerpenoids [48] (*Sargassum siliquastrum*), dihydroxystyrene metabolites [49] (association of* Poecillastra wondoensis* and* Jaspis* sp.), sesterterpenoids [50] (*Sarcotragus* sp.), sesterterpene sulfates [51] (*Dysidea *sp.), hyrtiosin B [52] (*Hyrtios *sp.), sargachromanols [53] (*Sargassum siliquastrum*), scalarane sesterterpenes [54] (*Hyatella *sp.), suvanine salt [55] (*Coscinoderma *sp.), bahamaolides A [56] (*Streptomyces *sp.), beta-carboline alkaloid [57] (*Synoicum *sp.), sphingolipid [58] (*Spirastrella abata*), and tris-aromatic furanones [59] (*Synoicum *sp.). Their IC_50_ are 50–95 *μ*g/mL, 28.7 to >200 *μ*g/mL, 12.5–19.9 *μ*g/mL, 31.3–33.8 *μ*M, 89 *μ*M, 118.4–172.9 *μ*M, 40.8–55.3 *μ*M, 5–17 *μ*M, 10.8 *μ*M, 48.2–68.9 *μ*M, 2–87 *μ*M, and 7.62–10.36 *μ*M, respectively. Other compounds which are also included in this category are Mycenon [60] (*Mycena* sp.) that is discovered through* Neurospora crassa* and* Ricinus communis *ICL with IC_50_ of 5.2–7.4 *μ*M and also polyoxygenated diterpenes [61] (*Phorbas *sp.) that is discovered through (unspecified) ICL with weak inhibition LC_50_ of 55–140 *μ*g/mL.

### 2.4. Non-MTB ICL with Synthetic Compounds

Three most established inhibitors for ICL were synthetic compounds discovered through non-MTB ICL: itaconate [14], 3-nitropropionate [15], and 3-bromopyruvate [16] with inhibition constant *K*_*i*_ of 120, 120 and 3 *μ*M, respectively [11]. These inhibitors are analog of succinate (itaconate, 3-nitropropionate) and glyoxylate (3-nitropropionate). However, these inhibitors are not being developed into drug as they are toxic and inhibit some key metabolism enzymes at* in vivo* level. Itaconate was suspected to cause hypertonicity towards cat blood pressure [17] affecting the growth of rats [18]; 3-nitropropionate was found to cause neurotoxicity [19] whereas 3-bromopyruvate seems to be an energy blocker [20, 21]. Therefore, these inhibitors were often used as control experiments in ICL inhibitors studies only.

Besides, many synthetic compounds were assayed on* Candida albicans* ICL to test their inhibitory potential as well, for instance, hydroquinone derivatives [62], bromophenols [63], analog of indole-containing natural compounds [64], and brominated resorcinol dimer [65]. Their IC_50_ were 0.28–1.02 mM, 2.65 *μ*M, 75 *μ*M, and 28 *μ*M, respectively.

## 3. Conclusion and Future Prospect

The pace of research in ICL has increased ever since the solution of the MTB ICL crystal structure [5] in 2000. Since then, ICL fundamental research has showed its potential as a drug target for latent TB treatment. The outcome also showed evidence that ICL is a persistence factor for MTB [3, 5, 12] but remains safe to be targeted [9]. With sufficient level of confidence for ICL as a potential drug target, GlaxoSmithKline (in collaboration with Global TB Alliance) has performed the first ever high-throughput screening (HTS) in 2000 but has terminated in 2005 after screening about 900,000 compounds as the outcome was modestly successful [34, 68]. The second HTS was reported in 2004, which was a HTS services by the Tuberculosis Antimicrobial Acquisition and Coordinating Facility (TAACF, an anti-TB program established by National Institute of Health (NIH)). This HTS screening through ChemBridge library which consist of 100,997 compounds has ended in 2009 with modest success too [69, 70]. The third HTS against 465 types of traditional Chinese herbs was carried out in 2006 [30]. Since the era of ICL potential inhibitor screening started, articles and reports were released every year till present. In this paper, we have summarized most of the potential inhibitors found or investigated throughout these years in tabulate form (Table 1).

To look for more potential ICL inhibitors, generally, the research method can be divided into biological assay and* in silico* approach. Biological assay usually refers either to whole cell assay or enzymatic assay that is implemented in high-throughput screening [30, 68, 69]. Apart from biological assay,* in silico* approach of virtual screening or ensemble docking (namely, molecular dynamic simulation-enhanced virtual screening) could be integrated into the current screening strategy to further reduce the failure cost in lead identification stage. Ensemble docking is a rather established* in silico* approach in the field of computer aided drug design, yet to be applied in ICL studies. Unlike conventional virtual screening, ensemble docking is able to address the degree of freedom during molecular docking process and hence increase the chances of better hit for potential inhibitor. As a complementary approach, rational drug design too is able to contribute in lead identification as well as lead optimization. The former could be either used to newly design potential inhibitor or used to combine the features of potential inhibitors obtained from the biological or virtual screening; the latter could enhance the binding affinity of a potential inhibitor, in order to achieve balance score between IC_50_, drug likeliness, and drug delivery [35–45]. Combination of both rational drug design and modified HTS (ensemble docking) might be a better approach when compared with only either one method.

## Figures and Tables

**Table 1:** Summary of potential inhibitors for MTB and non-MTB isocitrate lyase (ICL).

Number	Published year	Inhibitor	Source	Description	Target ICL	Inhibition^*^ (IC_50_)	Remarks
1	1977	Itaconate [11, 14] 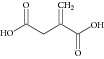	Synthetic	Succinate analog	*Pseudomonas indigofera *	*K*_*i*_ = 120	Established inhibitor

2	1982	3-Nitropropionate [11, 15] 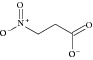	Synthetic	Succinate analog	*Pseudomonas indigofera *	*K*_*i*_ = 120	Established inhibitor

3	1990	Mycenon [60] 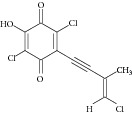	*Mycena* sp.	Fungi	*Acinetobacter calcoaceticus* *Neurospora crassa* *Ricinus communis *	5.2 *μ*M 7.4 *μ*M	No information on positive control

4	1990	3-Bromopyruvate [11, 16] 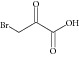	Synthetic	Glyoxylate analog	*Escherichia coli *	3 *μ*M	Established inhibitor

5	2005	DNAzyme [41]	Synthetic	—	*Mycobacterium tuberculosis *	—	

6	2006	Extract of traditional Chinese medicine [30]	*Zingiber officinale,* *Illicium verum *	Plant	*Mycobacterium tuberculosis *	47.7 μg/mL 18.2 μg/mL	Positive control IC_50_ of itaconate = 90 *μ*g/mL (good inhibitory)

7	2007	Hydroquinone derivatives [62] 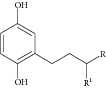	Synthetic	—	*Candida albicans *	0.28–1.02 mM	Positive control IC_50_ of itaconate = 0.06 mM (weak inhibitory)

8	2007	Halisulfate 1 [46, 47] 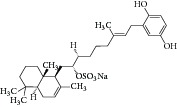	*Hippospongia *sp.	Marine sponge	*Magnaporthe grisea *	12.6 *μ*M	No information on positive control (high inhibitory)

9	2007	Bromophenols [27] 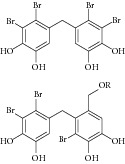	*Odonthalia corymbifera *	Red algae	*Magnaporthe grisea *	2.0–2.8 *μ*M	Positive control IC_50_ of 3-nitropropionate = 94.2 *μ*M (high inhibitory)

10	2008	Polyoxygenated diterpenes [61] 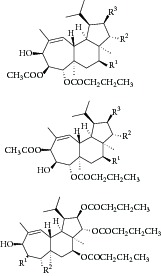	*Phorbas* sp.	Marine sponge	—	LC_50_ of 55–140 *μ*g/mL	No information on positive control (weak inhibitory)

11	2008	Meroditerpenoids [48] 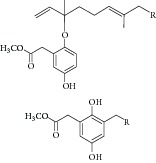	*Sargassum siliquastrum *	Brown algae	*Candida albicans *	50–95 *μ*g/mL	No information on positive control (weak inhibitory)

12	2008	Dihydroxystyrene metabolites [49] 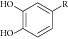	Association of *Poecillastra wondoensis* and *Jaspis* sp.	Marine sponge	*Candida albicans *	28.7 to >200 μg/mL	Positive control IC_50_ of itaconate = 5.8 *μ*g/mL (weak to moderate inhibitory)

13	2008	Sesterterpenoids [50] 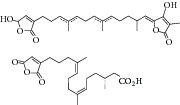	*Sarcotragus* sp.	Marine sponge	*Candida albicans *	12.5–19.9 *μ*g/mL	Positive control IC_50_ of Itaconate = 4.9 *μ*g/mL (moderate to high inhibitory)

14	2008	Sesterterpene sulfates [51] 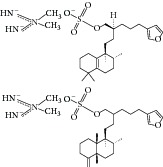	*Dysidea* sp.	Marine sponge	*Candida albicans *	31.3–33.8 *μ*M	Positive control IC_50_ of 3-nitropropionate = 50.7 *μ*M (high inhibitory)

15	2009	Pthalazinyl derivatives [35] 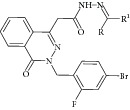	Synthetic	—	*Mycobacterium tuberculosis *	45–61% inhibition at 10 *μ*M	Positive control 3-nitropropionate has 63.2% inhibition at 100 *μ*M

16	2009	Hyrtiosin B [52] 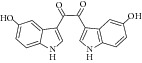	*Hyrtios* sp.	Marine sponge	*Candida albicans *	89 *μ*M	Positive control IC_50_ of 3-nitropropionate = 50.7 *μ*M (high inhibitory)

17	2010	Phthalazin-4-ylacetamides [36] 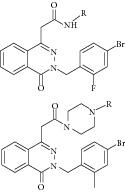	Synthetic	—	*Mycobacterium tuberculosis *	40.62–66% inhibition at 10 *μ*M	Positive control 3-nitropropionate has 68.2% inhibition at 100 *μ*M

18	2010	Extract of traditional Chinese medicine (I2906) [31] 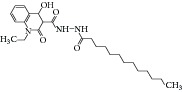	—	Plant	*Mycobacterium tuberculosis *	134 *μ*g/mL	Control samples were untreated samples

19	2010	5-Nitro-2-furoic acid hydrazones with furan-2-carbaldehyde [37] 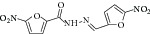	Synthetic	—	*Mycobacterium tuberculosis *	86.8% inhibition at 10 mM	Positive control 3-nitropropionate has 63.2% inhibition at 100 *μ*M (good inhibitory)

20	2010	Bromophenols [63] 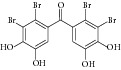	Synthetic	—	*Candida albicans *	2.65 *μ*M	Positive control IC_50_ of 3-nitropropionate = 50.7 μM (high inhibitory)

21	2010	5-Nitro-2,6-dioxohexahydro-4-pyrimidinecarboxamides [38] 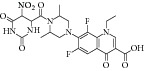	Synthetic	—	*Mycobacterium tuberculosis *	45.7% inhibition at 10 mM	Positive control 3-nitropropionate has 68.2% inhibition at 100 *μ*M (good inhibitory)

22	2010	Indole-containing natural compound (analog) [64] 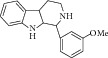	Synthetic	—	*Candida albicans *	75 *μ*M	Positive control IC_50_ of 3-nitropropionate = 50 *μ*M (high inhibitory)

23	2010	Isatinyl thiosemicarbazones derivatives [39] 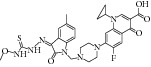	Synthetic	—	*Mycobacterium tuberculosis *	63.44% inhibition at 10 mM	Positive control 3-nitropropionate has 65.9% inhibition at 100 mM (good inhibitory)

24	2011	Brominated resorcinol dimer [65] 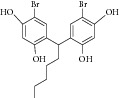	Synthetic	—	*Candida albicans *	28 *μ*M	Positive control IC_50_ of 3-nitropropionate = 6.0 *μ*M (good inhibitory)

25	2011	Sargachromanols [53] 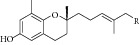	*Sargassum siliquastrum *	Brown algae	*Candida albicans *	118.4–172.9 *μ*M	Positive control IC_50_ of 3-nitropropionate = 34.8 *μ*M (moderate inhibitory)

26	2011	Scalarane sesterterpenes [54] 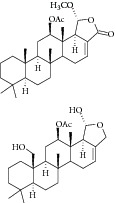	*Hyatella *sp.	Marine sponge	*Candida albicans *	40.8–55.3 *μ*M	Positive control IC_50_ of 3-nitropropionate = 27.9 *μ*M (weak inhibitory)

27	2011	Suvanine salt [55] 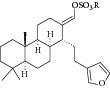	*Coscinoderma* sp.	Marine sponge	*Candida albicans *	5–17 *μ*M	Positive control IC_50_ of 3-nitropropionate = 27.9 *μ*M (moderate inhibitory)

28	2011	Chelerythrine extract [32]	*Chelidonium majus *	Plant	*Mycobacterium tuberculosis *	Expression level decreased 5 fold	—

29	2011	Mannich base, Ydcm67 [42] 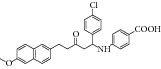	Synthetic	—	*Mycobacterium tuberculosis *	57.4% inhibition at 0.05 mg/mL	Positive control oxalic acid has 95.55% inhibition at 0.05 M

30	2011	Peptide inhibitor [43]	Synthetic	—	*Mycobacterium tuberculosis *	Inhibition rate 38.2–47.92%	Samples contain no peptide inhibitor or ICL in reaction system as controls

31	2011	3-Nitropropionamides derivatives [40] 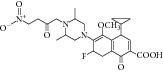	Synthetic	—	*Mycobacterium tuberculosis *	0.1 *μ*M	Positive control IC_50_ of 3-nitropropionate = 116.0 *μ*M (good inhibitory)

32	2012	Pyruvate-isoniazid analog with their copper complex [45] 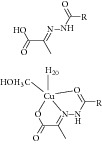	Synthetic	—	*Mycobacterium tuberculosis *	Inhibition rate 6–92%	Control docking using pyruvic acid

33	2012	Bahamaolides A (macrolide) [56] 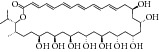	*Streptomyces* sp.	Actinomycete (Actinobacteria)	*Candida albicans *	10.8 *μ*M	Positive control IC_50_ of 3-nitropropionate = 20.1 *μ*M (high inhibitory)

34	2012	Beta-carboline alkaloid [57] 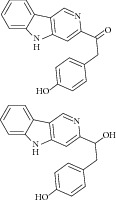	*Synoicum* sp.	Ascidian (sea squirt)	*Candida albicans *	48.2–68.9 *μ*M	Positive control IC_50_ of 3-nitropropionate = 38.6 *μ*M (weak inhibitory)

35	2012	Sphingolipid [58] 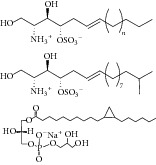	*Spirastrella abata *	Marine sponge	*Candida albicans *	2–87 *μ*M	Positive control IC_50_ of 3-nitropropionate = 1.0 *μ*M (weak to moderate inhibitory)

36	2012	Thio benzanilide [66] 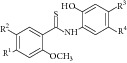	Synthetic	—	*Mycobacterium tuberculosis *	21–23% inhibition at 10 *μ*mol/L	Positive control 3-nitropropionate has 25% inhibition at 10–100 *μ*mol/L

38	2012	Salicylanilide derivatives [67] 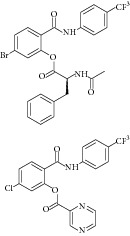	Synthetic	—	*Mycobacterium tuberculosis *	22–59% inhibition at 10–100 *μ*mol/L	Positive control 3-nitropropionate has 25–67% inhibition at 10–100 *μ*mol/L

39	2013	Tris-aromatic furanones [59] 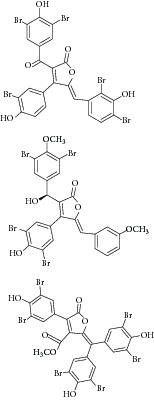	*Synoicum *sp.	Ascidian (sea squirt)	*Candida albicans *	7.62–10.36 *μ*M	Positive control IC_50_ of 3-nitropropionate = 13.91 *μ*M (Good inhibitory)

40	2013	Heptapeptide [44]	Synthetic	—	*Mycobacterium tuberculosis *	126 *μ*M	Positive control IC_50_ of 3-nitropropionate = 50.7 *μ*M (good inhibitory)

^*^Inhibition default unit is IC_50_ unless other units are mentioned in the table.

^*^Inhibitory potential of potential inhibitors was evaluated by the respective authors.
